# The Temporal Profile of Circulating miRNAs during Gestation in Overweight and Obese Women with or without Gestational Diabetes Mellitus

**DOI:** 10.3390/biomedicines10020482

**Published:** 2022-02-18

**Authors:** Anja Elaine Sørensen, Mireille N. M. van Poppel, Gernot Desoye, David Simmons, Peter Damm, Dorte Møller Jensen, Louise Torp Dalgaard, The DALI Core Investigator Group

**Affiliations:** 1 Department of Science and Environment, Roskilde University, 4000 Roskilde, Denmark; ltd@ruc.dk; 2 Faculty of Environmental and Regional Sciences and Education, Institute of Human Movement Science, Sport and Health, University of Graz, 8010 Graz, Austria; mireille.van-poppel@uni-graz.at; 3 Department of Obstetrics and Gynecology, Medical University of Graz, 8036 Graz, Austria; gernot.desoye@medunigraz.at; 4 Center for Pregnant Women with Diabetes, Department of Obstetrics, Rigshospitalet, 2100 Copenhagen, Denmark; pdamm@dadlnet.dk; 5 Macarthur Clinical School, School of Medicine, Western Sydney University, Campbelltown, NSE 2560, Australia; da.simmons@westernsydney.edu.au; 6 Department of Clinical Medicine, University of Copenhagen, 2200 Copenhagen, Denmark; 7 Department of Gynecology and Obstetrics, Odense University Hospital, 5000 Odense, Denmark; dorte.moeller.jensen@rsyd.dk; 8 Department of Clinical Research, Faculty of Health Sciences, University of Southern Denmark, 5000 Odense, Denmark; 9 Steno Diabetes Center Odense, Department of Gynecology and Obstetrics, Odense University Hospital, 5000 Odense, Denmark

**Keywords:** gestational diabetes mellitus, microRNA, circulating biomarkers, serum, temporal expression profile, miR-29a-3p

## Abstract

Circulating non-coding microRNAs (miRNAs) are important for placentation, but their expression profiles across gestation in pregnancies, which are complicated by gestational diabetes mellitus (GDM), have not been fully established. Investigating a single time point is insufficient, as pregnancy is dynamic, involving several processes, including placenta development, trophoblast proliferation and differentiation and oxygen sensing. Thus, the aim of this study was to compare the temporal expression of serum miRNAs in pregnant women with and without GDM. This is a nested case-control study of longitudinal data obtained from a multicentric European study (the ‘DALI’ study). All women (*n* = 82) were overweight/obese (BMI ≥ 29 kg/m^2^) and were normal glucose tolerant (NGT) at baseline (before 20 weeks of gestation). We selected women (*n* = 41) who were diagnosed with GDM at 24–28 weeks, according to the IADPSG/WHO2013 criteria. They were matched with 41 women who remained NGT in their pregnancy. miRNA (miR-16-5p, -29a-3p, -103-3p, -134-5p, -122-5p, -223-3p, -330-3p and miR-433-3p) were selected based on their suggested importance for placentation, and measurements were performed at baseline and at 24–28 and 35–37 weeks of gestation. Women with GDM presented with overall miRNA levels above those observed for women remaining NGT. In both groups, levels of miR-29a-3p and miR-134-5p increased consistently with progressing gestation. The change over time only differed for miR-29a-3p when comparing women with GDM with those remaining NGT (*p* = 0.044). Our findings indicate that among overweight/obese women who later develop GDM, miRNA levels are already elevated early in pregnancy and remain above those of women who remain NGT during their pregnancy. Maternal circulating miRNAs may provide further insight into placentation and the cross talk between the maternal and fetal compartments.

## 1. Introduction

During pregnancy, metabolic, physiological and immunological changes occur naturally to accommodate the development of the fetus and the placenta. A healthy pregnancy is characterized by transient insulin resistance and hyperlipidemia, among other changes, resulting in increased insulin secretion to secure normal glucose tolerance. However, genetic predisposition, pre-existing obesity, excessive gestational weight gain and maternal over-nutrition can result in a relatively impaired β-cell function and lead to hyperglycemia, deteriorated glucose tolerance and gestational diabetes mellitus (GDM) [1]. 

The importance of small non-coding post-transcriptionally regulatory microRNAs (miRNAs) in placentation and embryogenesis has been demonstrated using both in vitro models as well as in vivo rodent models. Their key role for a successful pregnancy was highlighted by studies in which an important miRNA-processing gene, Dicer1, was deleted in mice, resulting in embryonic developmental arrest and lethality [2]. 

The expression of selected miRNAs, such as miRNAs within the C14- and C19- miRNA cluster, as well as the miR-29a cluster, are different between first and third trimester placentas, suggesting a dynamic and development-dependent expression of individual miRNAs [3]. The first trimester placenta dominantly expresses miRNAs reflective of angiogenic, immune suppressive and tissue remodeling characteristics, while term placentas express miRNAs linked to immunological and hematopoietic activities [3]. Additionally, both circulating miRNAs [4] and placenta-derived exosomes, potential carriers of miRNAs, increase continuously during gestation [5]. Lastly, miRNAs have been shown to modulate and partake in processes and pathways important for successful placentation and placental homeostasis, such as fine-tuning the maternal immune system through immune-tolerogenic regulation, proper differentiation and proliferation of trophoblasts, as well as remodeling the spiral arteries and oxygen sensing (reviewed in [6]).

It is not known whether changes in miRNAs in the circulation precede the onset of metabolic changes associated with GDM, thus potentially contributing to its pathogenesis, or whether circulating miRNA changes in GDM are downstream events secondary to GDM pathology. Here, we compare longitudinal changes of selected miRNAs circulating in obese normal glucose tolerant women versus women who develop GDM. miRNAs were chosen for this study based on either previous findings in GDM (miR-16, -29a, -103, -122, -134, -223 and -330 [4,7,8,9,10,11,12,13,14,15,16,17,18]), impaired metabolism (miR-16, -29a, -122, -103 and -223 [10,19,20,21]) or an association with or expression in the placenta (miR-29a, -134 and -433 [3,22]). Establishing the temporal expression profile of circulating miRNAs associated with GDM could improve our understanding of processes important for placenta homeostasis and GDM and may, in the future, provide a basis for preventive treatment. 

## 2. Materials and Methods

### 2.1. Study Participants

This nested, temporal, observational, case-control study comprised 41 obese women with singleton pregnancy complicated by GDM as defined according to the IADPSG/WHO2013 criteria (oral glucose tolerance test (OGTT), venous plasma glucose: fasting ≥ 5.1 mmol/L, 1 h  ≥  10 mmol/L, 2 h  ≥  8.5 mmol/L—at least one abnormal value) at 24–28 weeks. The 41 women with GDM were randomly selected from all women in the lifestyle study who were diagnosed with GDM at 24–28 weeks. As controls we included 41 age- and BMI-matched obese women with singleton pregnancy who remained normal glucose tolerant (NGT) during their pregnancy. All the women were originally included in the European, multicenter, randomized ‘Vitamin D and lifestyle intervention for GDM prevention (DALI)’ trial and were followed at baseline (≤19 ± 6 days of gestation) and at 24–28 and 35–37 weeks of gestation [23]. There were no significant differences in terms of the type of interventions in each group (Pearson chi-square test, two-sided, *p* = 0.48). Women who developed GDM after baseline received standard care according to local guidelines. The study was approved by the relevant local ethics committees and was performed according to the Declaration of Helsinki II. The trial registration was ISRCTN70595832. Informed written consent was obtained from all subjects involved in the study at baseline.

### 2.2. Sample Collection and Storage

At baseline, women underwent assessments, and fasting blood samples were collected prior to and during an OGTT. This was repeated at 24–28 weeks and at 35–37 weeks. From women with GDM at 24–28 weeks based on locally measured glucose values (*n* = 16), only fasting blood samples were obtained at 35–37 weeks, as previously described [23,24]. Thus, out of the 41 women with GDM (based on centrally measured values), 25 women were not diagnosed/treated at 24–28 weeks. Therefore, glucose values were also available at 35–37 weeks. The whole blood samples were separated into serum and stored at −20 °C or −80 °C to be further handled in the ISO-certified central trial laboratory in Graz, Austria.

### 2.3. Isolation and Analysis of miRNA

The temporal expression profile of eight small non-coding miRNAs was measured in serum (miR-16-5p, -29a-3p, -103-3p, -134-5p, -122-5p, -223-3p, -330-3p and miR-433-3p).

Circulating total RNA was extracted from 250 µL of serum using TRI^®^ Reagent LS (Sigma-Aldrich, Søborg, Denmark) according to the manufacturer’s protocol and with the addition of synthetic ath-miR-159 (0.25 picomol) as a spike-in control and glycogen (15 nmol) as a carrier. Quality control of selected RNA samples was performed using Agilent Small RNA chips on the Agilent 2100 Bioanalyzer (Agilent Technologies, Glostrup, Denmark).

A total of 100 ng of RNA was reverse transcribed using miRNA-specific stem-loop RT primers (Supplementary Table S1) and a MultiScribe™ MicroRNA Reverse Transcription kit (ThermoFisher Scientific, Slangerup, Denmark) according to the manufacturer’s protocol, with the addition of synthetic spike-in control Caenorhabditis elegans (cel)-miR-39 (0.5 attomol) during cDNA synthesis. The mix was incubated at 16 °C for 30 min, 42 °C for 30 min and 85 °C for 5 min. Subsequently, real-time quantification was performed using the ViiA real-time PCR System (ThermoFisher Scientific, Slangerup, Denmark) detection system. The PCR reaction included diluted cDNA with appropriate primers and QuantiTect SYBR Green PCR master mix (Qiagen, Copenhagen, Denmark). The 384-well plates contained an interplate calibrator consisting of pooled sample material. All the reactions were run in duplicates. The raw C_T_ data were determined using the default threshold setting. The mean C_T_ values were normalized against the geometric mean of three reference genes: the two added spike-ins (cel-miR-39 and ath-miR-159) and the endogenous small nuclear U6. Using the standard curve method, miRNA quantities were extrapolated and the GDM-to-NGT ratios of the serum miRNAs were calculated. 

### 2.4. Statistical Analysis

A temporal analysis of miRNA levels was performed using linear mixed models and the generalized least squares ‘gls’-function in the R-based nlme-package (vers. 3.1-152) [25] on log-transformed data. Individual time points and groupings were coded as categorical variables, and an unstructured covariance pattern was applied to account for the correlations in data over time. Estimates for both groups (NGT or GDM), time point (baseline, week 24–28 or 35–37) and the interaction between group and time point were obtained. Continuous baseline data were analyzed by an independent t-test if data were normally distributed or were assessed by a Shapiro–Wilk test or Mann–Whitney test if the data were not normally distributed. Categorical data were analyzed by a chi-square test (2-sided). Correlation at the individual time point was assessed using a Pearson correlation coefficient and visualized by the corr-plot package (vers. 0.89) [26]. The association between miRNA levels and changes in clinical variables was assessed by a linear regression. Gestational weight gain (GWG) was defined as the weight (in kg) gained from baseline. Predicted target genes for the individual miRNAs were identified using TargetScan (v.7.2, human, http://www.targetscan.org/vert_72/, accessed on 20 May 2021) [27], and pathway enrichment analysis was performed using the PANTHER (Protein ANalysis THrough Evolutionary Relationships) Classification System (v.15.0, http://pantherdb.org/, accessed on 20 May 2021) and the PANTHER pathways annotation set [28]. The enrichment analysis was visualized by RStudio (v. 1.3.1093, RStudio, PBC Boston, MA, USA, http://www.rstudio.com) using the ggplots2 (v.3.1.0, https://ggplot2.tidyverse.org/) package [29].

An in silico analysis of placenta-enriched genes as potential targets of circulating miRNAs was based on two generated lists, one for all predicted targets of the selected miRNAs and one for all highly enriched genes present in the placenta according to the Human Protein Atlas, accessed on 20 May 2021 [30]. The statistical significance of the overlap between these two lists was tested using the exact hypergeometric probability described in [31].

An a priori power analysis was conducted. With a minimal acceptable power of 85%, an SD of 30% and a chance of 50%, a minimum of 32 subjects per group were needed with the significance criterion set to α = 0.05. 

All analyses were conducted using SPSS, version 27 (SPSS Inc., Chicago, IL, USA) or RStudio (v. 1.3.1093, RStudio, PBC Boston, MA, USA, http://www.rstudio.com). GraphPad Prism version 9 (GraphPad Inc., La Jolla, CA, USA) was used for graphical presentation. Statistical significance was defined as *p*  <  0.05.

## 3. Results

### 3.1. Anthropometric Measurement of Study Participants at Baseline 

At baseline, women who were later diagnosed with GDM at 24–28 weeks already had higher fasting and 2 h glucose levels compared with women who remained NGT in pregnancy (Table 1). 

### 3.2. Temporal Changes in Clinical and Biochemical Parameters during Gestation

The temporal changes in the clinical and biochemical variables for both groups can be found in Supplementary Figure S1A–P. In brief, gestational weight gain (GWG) was similar between the two groups (Supplementary Figure S1A), while those women who developed GDM presented with a significant increase in fasting plasma glucose, as well as 1 h post-OGTT glucose levels, from baseline to the time of GDM diagnosis (Supplementary Figure S1C,D). Similarly, 2 h post-OGTT insulin levels increased more from baseline to 35–37 weeks in the women with GDM compared with the NGT women (Supplementary Figure S1H). Significant interactions between the groups over time were observed for HDL levels, which overall decreased from baseline to week 35–37 (Supplementary Figure S1L). 

### 3.3. The Temporal Profile of miRNAs during Pregnancy

**Figure 1:**
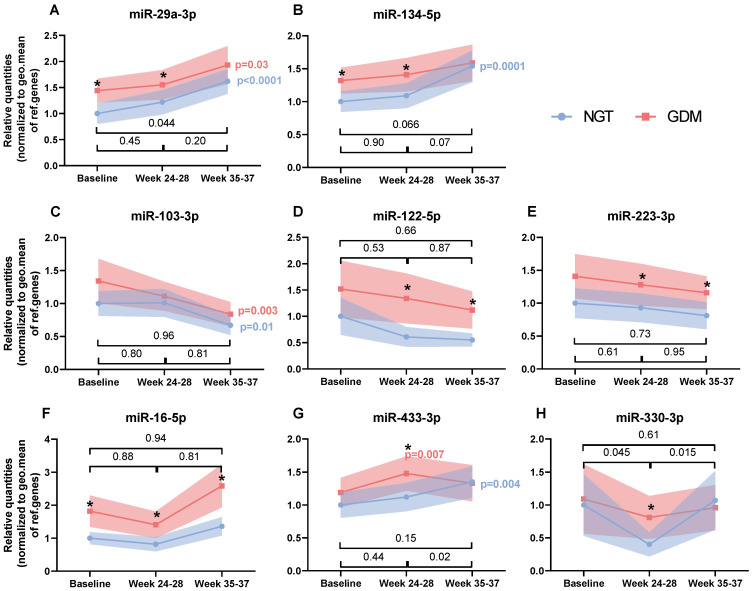
Temporal profile of circulating miRNAs during pregnancy grouped by the pattern of the changes (**A**–**H**). Relative mean quantities with 95% CI of serum microRNAs expressed in women with either normal glucose tolerance (NGT) or women who developed gestational diabetes mellitus (GDM). The data were normalized against the geometric mean of snRNA U6, ath-miR-159 and c.el.miR-39. All indicated *p*-values were determined by linear mixed models on logarithmic transformed data including maternal age and BMI as covariates. *p*-values in colors describe the changes since baseline in either NGT or GDM. *p*-values in black describe the interaction between the groups and time. * denotes a significant difference between the two groups at baseline, at 24–28 weeks or at 35–37 weeks of gestation.

The changes in mean miRNA levels during gestation between the two groups are illustrated in Figure 1. In general, they followed three distinct patterns: Continuous increase, continuous decrease and peak or nadir at 24–28 weeks.

The levels of miR-29a-3p (Figure 1A) and miR-134-5p (Figure 1B) increased consistently over gestation (from baseline to 35–37 weeks) in both groups. The changes over time in miR-29a-3p were greater in the NGT women (NGT: 62%, *p* < 0.0001 vs. GDM: 34%, *p* = 0.03). A comparison of the trajectory for miR-29a-3p between the two groups showed that changes in the miR-29a-3p levels over time were substantially different (*p* = 0.044, baseline vs. 35–37 weeks) (Figure 1A). Similarly, the baseline levels of miR-134-5p increased by 54% in the NGT women (*p* = 0.0001) while a slightly smaller (20%, *p* = 0.13) increase was observed in the GDM women, although the mean changes over time did not differ significantly between the groups (baseline vs. 24–28 weeks, *p* = 0.9; baseline vs. 35–37 weeks, *p* = 0.066).

Moreover, levels of miR-103-3p (Figure 1C), miR-122-5p (Figure 1D) and miR-223-3p (Figure 1E) decreased during gestation. In both groups, miR-103-3p changed significantly with time (NGT: −33%, *p* = 0.01; GDM: −37%, *p* = 0.003; baseline vs. 35–37 weeks), although no significant interaction between the two groups and time was observed. Baseline miR-122-5p levels decreased by 39% in NGTs and by 12% in GDM at the second time point. No significant changes were observed from the second to the third time point in the individual groups nor did miR-122 levels change significantly differently between the two groups. Despite a total decrease of almost 20% in the miR-223-3p levels, no significant changes were observed within the individual groups or between the groups over time.

MiR-16-5p displayed a different pattern (Figure 1F). At first, miR-16-5p decreased in both groups (NGT: −18%, *p* = 0.05; GDM: −23%, *p* = 0.03), but later miR-16-5p increased to even higher levels than those observed at baseline (NGT: 36%, *p* = 0.15; GDM: 42%, *p* = 0.14). As reported earlier, baseline levels of miR-16-5p were significantly different between the two groups. However, the mean change over time from baseline to 35–37 weeks was not different between the two groups.

Unlike miR-16-5p, miR-433-3p (Figure 1G) first increased in both groups. This initial increase was only significant in the GDM women (*p* = 0.007). This was then followed by an additional increase in NGT (from 12% to a total of 35%, *p* = 0.004, baseline vs. 35–37 weeks) or a slight decrease in GDM (from 24% to 12%, *p* = 0.38, baseline vs. 35–37 weeks). Still, the confidence intervals were overall wide and no significant differences between the two groups were observed.

Interestingly, levels of miR-330-3p (Figure 1H) in the NGT group first decreased from baseline to 24–28 weeks (*p* = 0.019) and then increased until 35–37 weeks (*p* = 0.16). During the same period, miR-330-3p did not change (*p* = 0.64 and *p* = 0.48, respectively) in the GDM women. The changes over time in mean level of miR-330-3p were significantly different between the two groups (from baseline vs. 24–28 weeks, *p* = 0.045).

### 3.4. Correlations between Clinical Values

**Figure 2:**
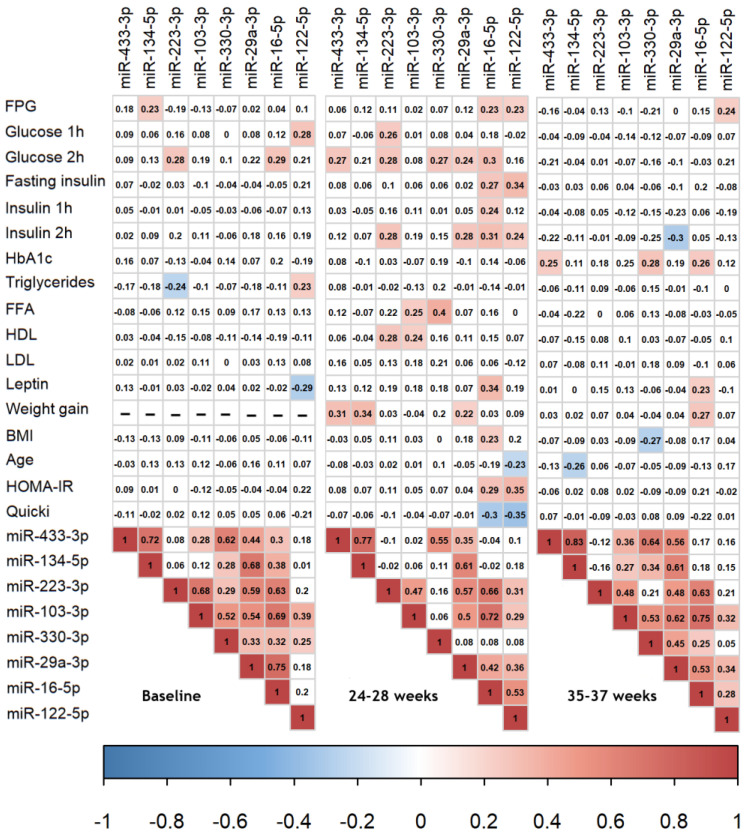
Correlation between the selected miRNAs and the clinical or biochemical parameters at each separate time point. Color intensity is proportional to the Pearson correlation coefficient. Positive correlations are displayed in red and negative correlations are displayed in blue. Colored correlations are significant at *p* < 0.05. No gestational weight gain was present at baseline, marked here with ‘-’. At 35–37 weeks, the correlations between miRNA and values pertaining to the OGTT (here, 1 h and 2 h glucose and insulin) are based on women with GDM, based on centrally measured glucose values (*n* maximum = 25) and women with NGT. The levels of miRNAs were normalized against the geometric mean of snRNA U6, ath-miR-159 and c.el.miR-39 and logarithmically transformed prior to analysis, as were the selected clinical variables, according to the methods section. All correlation plots can be seen in Supplementary Figure S2A–C.

Pregnancy is a dynamic process that showed initial increases in some circulating biochemical variables (e.g., 1 h and 2 h post-OGTT glucose and insulin, HOMA-IR, LDL cholesterol, triglycerides and HDL cholesterol) while others decreased (e.g., FFA) or remained at similar levels from baseline to 24–28 weeks of gestation (e.g., HbA1c). In the third trimester, some variables did not change further (e.g., HOMA-IR and LDL cholesterol) while others either increased (e.g., HbA1c and FFA) or decreased (e.g., fasting plasma glucose (FPG) and HDL cholesterol). This prompted us to analyze the correlations between the selected miRNAs and clinical variables separately at the three different time points (Figure 2, baseline, 24–28 weeks and 35–37 weeks). The strength of the significant correlations between the miRNAs and clinical variables at the different time points was generally weak to modest (Figure 2 and Figure S2A–C). All miRNAs but one (miR-103-3p) correlated positively with either glucose- or insulin-dependent variables at one time during gestation. Gestational weight gain was positively associated with miR-433-3p, -134-5p, -29a-3p and miR-16-5p at either week 24–28 or week 35–37. 

As expected, the majority of the miRNAs correlated positively with each other at all time points. 

### 3.5. Gestational Weight Gain Is Associated with miR-134-5p and miR-433-3p Levels

Excessive gestational weight gain is associated with adverse maternal outcomes, such as cesarean deliveries and pre-eclampsia, and neonatal outcomes, such as large-for-gestational-age infants. Weight gain in the first trimester is attributed to the expansion of blood volume in the pregnant woman and to early placental development and not as much to fat accrual [32]. This promoted us to investigate whether weight gain from baseline to 24–28 weeks influenced the miRNA levels measured at this time point. Seven of the eight miRNAs displayed a significant difference between the two groups at 24–28 weeks of gestation (Figure 1A,B,D–H). Among these, miR-433-3p, -134-5p, -29a-3p and -16-5p correlated positively with gestational weight gain. In the linear regression analysis, which was adjusted for gestational age, the maternal pre-pregnancy BMI and miRNA levels at baseline the levels of both miR-433-3p and miR-134-5p at 24–28 weeks remained positively associated with gestational weight gain from baseline to 24–28 weeks (Table 2). However, the changes in the levels of miR-29a-3p and miR-16-5p could not be explained by gestational weight gain. 

### 3.6. Impaired Glucose Metabolism and Dyslipidemia Is Associated with Selected miRNA

A linear regression analysis was carried out using various combinations of glucose-derived variables to further identify what associates with the expression of the individual miRNAs. We observed a positive association between miR-134-5p, measured at 35–37 weeks of gestation, and changes in HOMA-IR in women both with and without GDM (Table 3).

Besides hyperglycemia, dyslipidemia during pregnancy has been associated with adverse pregnancy outcomes. Hence, changes in lipid profiles could be reflected in miRNA levels. At the time of GDM diagnosis, no apparent relationship was observed between the changes in HDL cholesterol, LDL cholesterol or triglycerides from baseline to 24–28 weeks of gestation and miRNA levels, with the exception of miR-103-3p (Table 4).

### 3.7. Pathway Analysis 

**Figure 3:**
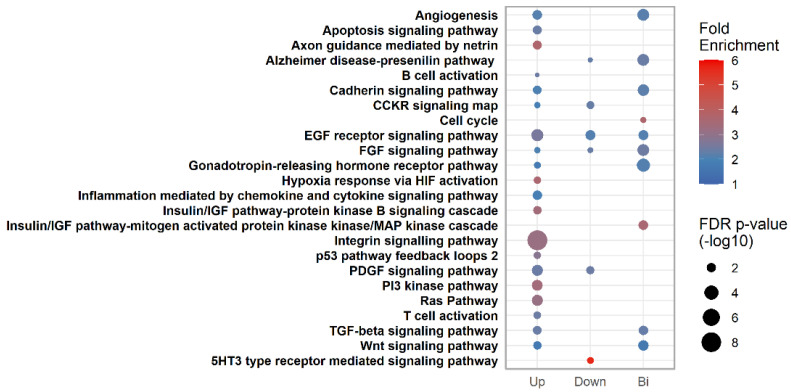
Pathway enrichment analysis of the predicted targets of the three defined miRNA groups. The bubble fill color represents the degree of enrichment, while bubble size represents the FDR-corrected *p*-value on a −log10 scale. MiRNAs displaying an overall increasing (Up: miR-29a-3p, -134-5p and -433-3p), decreasing (Down: miR-122-5p, -103-3p and -223-3p) or a bimodal (Bi: miR-16-5p and miR-330-3p) longitudinal expression pattern were grouped together. CCKR: Cholecystokinin Receptor, EGF: Epidermal Growth Factor, FGF: Fibroblast Growth Factor, HIF: Hypoxia-inducible Factor, IGF: Insulin Growth Factor, PDGF: Platelet-Derived Growth Factor, TGF: Transforming Growth Factor, Wnt: Wingless-type, 5HT3: Serotonin receptor, PI3: Phosphoinositide-3.

In order to elucidate which potential biological pathways could be regulated by the investigated miRNAs, three groups were constructed based on similar temporal profiles in the total study population (*n* = 82) upon the mixed model analysis. The predicted targets of these miRNAs were subjected to a pathway enrichment analysis (Figure 3).

When the 3 miRNAs (miR-29a-3p, -134-5p and -433-3p) with gradually increasing levels were combined, they were predicted to target 1710 unique transcripts, with the majority of these belonging to miR-29a-3p. A total of ten different pathways was found to be enriched. For instance, transcripts affected by any of these three miRNAs are involved in immunological and insulin-relevant pathways, as well as metabolic adaptation to hypoxia and integrin signaling. 

On the other hand, miR-122-5p, -103-3p and -223-3p decreased gradually and targeted a total of 1354 unique transcripts. The 5HT3-type receptor-mediated signaling pathway, which has associations to obesity and diabetes [33], showed the highest enrichment and was solely represented by transcripts targeted by the miRNAs with a decreasing temporal profile.

Although the remaining two miRNAs, miR-16-5p and miR-330-3p, had bi-modal temporal expression levels during pregnancy, they were found to potentially interact with the highest number of transcripts (*n* = 2301).

Among all three groups, two pathways associated with glucose regulation, EGF receptor signaling and FGF signaling showed shared enrichment, most likely because they include genes with antagonistic effects within the same pathway.

The placenta separates maternal and fetal circulation. miRNAs may facilitate placental communication with the maternal and fetal compartments, reviewed in [34]. An in silico comparison between human placental enriched genes (*n* = 494) derived from antibody-based protein profiling of healthy placenta tissue and all uniquely predicted targets (*n* = 3322) of the eight selected miRNAs demonstrated an overlap of 84 genes (hypergeometric overlap statistics *p* < 10^−30^, Supplementary Figure S3). Using these 84 overlapping genes in an enrichment analysis, the gene ontology (GO) biological processes related to development were among the highly enriched GO terms. Thus, abnormal changes in maternal circulating miRNAs could potentially lead to placenta maldevelopment and, hence, dysfunction. Of note, out of the selected eight miRNAs, miR-433-3p and miR-134-5p belong to the C14 miRNA cluster, which is located at the imprinted DLK-DIO3 region and is predominately expressed in placenta tissue. Hence, any increases in these two miRNAs could reflect increases in placenta weight. However, we observed no differences in placenta weight (630 g versus 620 g, *p* = 0.80) between the NGT and GDM women, and none of the miRNAs correlated positively at either time point with placental weight. 

## 4. Discussion

In this study, the temporal profile of selected circulating miRNAs in pregnancy was evaluated prior to 20 weeks of gestation and at 24–28 and 35–37 weeks of gestation in overweight/obese women with normal glucose tolerance and pregnancies affected by GDM. We have previously reported differences between women with GDM and those who remained NGT in the selected miRNAs at baseline [7]. All of the investigated miRNAs had higher levels during pregnancy in the women who later developed GDM. All of these, except for miR-103-3p, were also different at 24–28 weeks of gestation, while levels of miR-122-5p, -223-3p and -16-5p were significantly higher in the GDM group by the third trimester. Half of the miRNAs changed over time either in both groups (miR-29a-3p and miR-103-3p) or only in the NGT group (miR-134-5p and -433-3p). Importantly, miR-29a-3p demonstrated a significant interaction between the two groups and time. Additionally, levels of clinical and biochemical parameters related to impaired glucose metabolism were concomitantly measured. Gestational weight gain was positively associated with miR-134-5p and miR-433-3p levels, while changes in HOMA-IR and HDL were associated with 134-5p and 103-3p, respectively. 

Most studies on circulating miRNAs in relation to GDM have only investigated the expression profile at a single time point in gestation [8,10,11,14,15,16,17,18,35,36,37,38,39,40,41,42], with the exception of one study [4]. There is limited consistency between the investigated miRNAs, although miR-16, -29a, -132, -223 and -330 are reported in more than one study.

Interestingly, four of the five miRNAs that were significantly increased during the second visit (24–28 weeks of gestation), miR-29a, miR-134-5p, miR-433-3p and miR-330-3p, are involved in pancreatic beta cell function [9,43,44,45,46,47]. MiR-29a, which increased with increasing glucose concentrations, impairs glucose-stimulated insulin secretion at least partially via targeting the exocytosis protein Syntaxin 1 [43,44,45], while miR-330-3p targets glucokinase, the gene responsible for Maturity Onset Diabetes of the Young, subtype 2 (MODY2). Both miR-134-5p and miR-433-3p belong to a microRNA cluster (C14MC) within the imprinted DLK1-DIO3 locus on chromosome 14. This locus has a role in placental and fetal development and is also important for beta cell function [47], although it should be added that only miR-433-3p has been directly tested for a role in beta cells [46]. Thus, the differential regulation of these four miRNAs in serum from women with GDM could, perhaps, also reflect beta cell dysfunction. In this respect, it is also noteworthy that a common pathway that is potentially regulated by the GDM-upregulated miRNAs (miR-29a, miR-134-5p and miR-433-5p) is integrin signaling (Figure 2). During gestation, beta-cell integrin signaling is important, because prolactin and placental lactogen increases the expression of integrin α6 to increase beta-cell replication [48]. Given that microRNA tissue-to-tissue crosstalk has been demonstrated [49], at least for miR-29a, it is an interesting hypothesis that increased levels of these miRNAs could mediate beta cell dysfunction, possibly via actions on integrin signaling.

We are able to confirm the findings by Cao and colleagues, who showed that plasma miR-16-5p progressively increased during pregnancy (measured at 16–20 weeks, 20–24 weeks and 24–28 weeks of pregnancy) in women diagnosed with GDM [4]. Similarly, high-throughput sequencing of serum samples collected at 16–19 weeks of pregnancy, followed by validation by qPCR, showed an upregulation of miR-16-5p in GDM women compared to pregnant women without GDM [39], which is also consistent with our results. Others did not observe any significant differences in serum miR-16-5p at an average of 21 weeks of gestation [16] or plasma miR-16 at gestational weeks 23–31 [11]. Likewise, levels of the blood leukocyte miR-16-5p measured in the third trimester (~gestational age 34) were similar between the control and GDM women [17]. In order to accommodate the increased metabolic demands of the fetus, the β-cell mass of pregnant woman undergoes an expansion. In mice, non-β-cells of unknown source(s) are recruited and contribute to the increase in β-cell numbers during pregnancy, and the number of neurogenin 3 (NGN3)-positive cells, as well as NGN3-expressing islets, decreases [50]. Interestingly, the NGN3 transcript is a validated target of miR-16-5p [51], and it could, at least in theory, explain how increased levels of miR-16-5p during gestation could lead to impaired glucose metabolism.

In early pregnancy (6-15 weeks of gestation), serum exosomal miR-29a-3p was found at higher levels in Canadian women with GDM [15], as were serum miR-29a-3p measured at 18–23 weeks of gestation in Mexican women with GDM [16] and serum levels measured prior to 20 weeks of gestation in European women with GDM in a previous report of the DALI study [7]. While these studies are consistent with current results, decreased serum miR-29a at 16–19 gestational weeks was found in Chinese women diagnosed with GDM based on the American Diabetes Association (ADA) criteria [8]. The observed discrepancy could be due to differences in GDM diagnostic criteria or ethnicity. 

Maternal pancreatic β-cell mass increases, as does the efficiency of glucose-stimulated insulin secretion, due to the rising levels of human placental lactogen and prolactin and as a response to insulin resistance in pregnancy. An initial increase in miR-433 levels was observed in both groups. However, after GDM diagnosis, miR-433 levels dropped in women with GDM, while miR-433 continued to increase in women with NGT. In a high-glucose environment in vitro, miR-433 was downregulated in insulin-secreting MIN6 cells, while the restoration of miR-433 levels through miR-mimics protected the β-cells [46]. As speculation, it is possible that miR-433 could contribute to the compensatory effects in an attempt to maintain glucose homeostasis and that this effect was blunted in the GDM group. Alternatively, the treatment of GDM could have influenced miR-433 levels.

During the pregnancy period, circulating miR-134-5p levels continued to increase in both groups. It does not appear that changes in glucose or insulin levels nor changes in insulin sensitivity or insulin resistance influenced the expression of miRNA levels in the third trimester, except for miR-134-5p. Interestingly, the induction of miR-134-5p by hyperglycemia in vitro resulted in impaired cell migration and tube formation abilities in endothelial cell progenitors [52]. Elevated levels of miR-134-5p are associated with both GDM [7,40] and pre-eclampsia and the suppression of infiltration of trophoblast cells [53], suggesting that dysregulated miR-134 could contribute to GDM pathology. We observed that both miR-433-3p and miR-134-5p were positively associated with gestational weight gain. This locus has been found important for fetal viability and placental development and function [54,55], as well as the predisposition to diabetes outside pregnancy [56]. With advancing gestational age, miRNAs within the C14MC increase from 4th to 16th weeks of gestation [57]. When primary first and third trimester trophoblastic cells were profiled, the expression of members of the C14MC cluster decreased from the first to third trimester trophoblasts when compared to miRNAs belonging to another placenta-associated miRNA cluster, i.e., C19MC [58]. The reason for the discrepancy between these observations could be that cell types other than trophoblasts contribute to the total circulating miR-134 and miR-433 pool.

Elevated serum miR-103 has been associated with HNF1A-MODY carriers [59], and a meta-analysis of multiple tissue types found an association between upregulated miR-103 and T2D [19]. In relation to our study, regardless of the time point investigated, miR-103-3p levels were higher in women with GDM, although no significant differences were observed at the individual time points. This is in accordance with two other studies measuring miR-103 before 20 weeks of gestation [7] or at 26–30 weeks of gestation [13]. Inhibition of miR-103 in obese mice improved insulin sensitivity and glucose homeostasis [60]. Lowering miR-103 using miRNA inhibitors could, thus, potentially be a treatment option for GDM. We observed that miR-103-3p progressively decreased in both groups and that miR-103 was inversely associated with changes in the LDL levels at the time of GDM diagnosis. 

miR-103-3p, -122-5p and -223-3p had predicted targets related to the 5HT3-type receptor-mediated signaling pathway. During pregnancy, glucose-stimulated insulin secretion in mouse pancreatic β-cells is partly controlled by the 5HT3-type receptor-mediated signaling pathway through simultaneous increases in serotonin levels [61]. The serotonin transporter (HTT, also known as SLC6A4) is a predicted target of miR-103-3p. Interestingly, infants born to mothers with GDM showed decreased methylation of placental *SLC6A4,* with resulting increased SLC6A4 mRNA levels [62]. The functional consequences of decreased miR-103, potentially on SLC6A4 mRNA levels, in GDM remain to be identified. 

Four studies have investigated miR-223, either in plasma [11,12,18] or serum [7], and consistently found higher miR-223 levels in GDM than in NGT pregnancies, at least in early gestation. Moreover, outside pregnancy, islet miR-223 is upregulated in subjects with diabetes. In vitro stimulation of MIN6 cells with high glucose or tumor necrosis factor α increased miR-223 levels [63]. We observed that, compared with the controls, the mean miR-223 levels increased at every time point measured during pregnancy. Women who develop GDM are often insulin-resistant, even before conception, which is often associated with maternal obesity. Insulin-resistant women displayed higher levels of adipose tissue miR-223 compared with non-insulin-resistant women [64], potentially explaining why circulating miR-223 levels were also elevated in our GDM group. Being a hematopoietic-cell–derived miRNA, miR-223 is highly involved in immune responses. A successful pregnancy depends on a shift towards an immune tolerant and anti-inflammatory state, in order to support the growing fetus. Thus, dysregulated miR-223 might contribute not only to impaired glucose metabolism but might also influence immune cells at the maternal–placental interface. 

Third-trimester miR-330-3p levels were found to be upregulated in two separate studies in lean women with GDM [13,14]. This is consistent with our study for a similar time point (24–28 weeks). Since most of our subjects were obese, higher miR-330-3p levels in GDM than in NGT appear to be independent of the BMI of the pregnant woman. A bimodal expression pattern was observed for miR-330-3p for unknown reasons. However, a target gene analysis demonstrated the post-transcriptional modulation of genes relevant for insulin secretion, as well as the differentiation and proliferation of β-cells by miR-330-3p [9,65,66]. It remains to be studied whether the bimodal miR-330-3p levels are related to the increasing insulin concentrations in women between weeks 24 and 30 [67].

The liver-enriched miR-122 progressively decreased during gestation in both the NGT and GDM groups but consistently showed higher levels in the subjects with GDM. Encapsulated within extracellular vesicles and measured early in gestation (between 6 and 15 weeks of gestation), serum miR-122 was significantly higher in women diagnosed with GDM compared with controls [15], in line with our study. Outside pregnancy, a higher degree of adiposity, inflammation, insulin resistance and triglycerides and lower HDL cholesterol were all associated with higher levels of circulating miR-122, especially in participants with either a diagnosis of T2D or metabolic syndrome [20]. Insulin resistance peaked in both groups around the time of diagnosis, and HOMA-IR correlated positively with miR-122 at this time point, strengthening the role of miR-122 as a marker of impaired metabolism. 

### Strengths and Weaknesses

The study population, although mostly obese, was largely of European descent. This reduces the potential confounding effects of ethnicity, which is a well-known risk factor of GDM. However, this also warrants validation studies in a more ethnically diverse population, as well as in lean women. A strength of the current study is its temporal nature, allowing us to investigate the changes over time within each woman of the two groups. It is worth mentioning that after GDM diagnosis appropriate treatment according to local guidelines was initiated. In our study, a lack of information on treatment modalities precluded a sub-analysis on whether treating GDM influenced the temporal miRNA profile, a question that warrants further study. The recruitment site was associated with GDM at baseline and at 24–28 weeks [68], and performing a sub-analysis including only women within the same recruitment site could have resulted in a more homogenous, but less representative, study population. Furthermore, by matching for maternal BMI and age, differences in insulin sensitivity between women with GDM and those who remained NGT might have been reduced. However, now, the differences in the temporal profiles of the miRNAs can be attributed to the impaired metabolism causing GDM, independent from changes due to higher BMI or age.

We employed a targeted miRNA profile approach. Thus, other potentially important miRNAs may have been overlooked. As of now, there is limited knowledge of the population baseline levels of circulating miRNAs in healthy individuals, let alone in pregnancy. Establishing such reference ranges would aid in translating and using miRNAs as diagnostic/prognostic markers in the clinic.

An interesting but open question is the origin of the circulating miRNAs. Recent advances within the field of sequencing (SLAM-seq [69]) using tissue-specific incorporation of 4-thiouridine (4-TU) could prove useful for in vivo tracking of miRNAs released into circulation. Despite miRNA levels demonstrating high correlations between repeated samples over a larger sampling period in healthy individuals [70,71], more longitudinal studies within pregnant women are needed should miRNAs be utilized in clinical practice. 

## 5. Conclusions

In summary, overweight/obese pregnant women who are later diagnosed with GDM present with miRNAs levels above those of obese pregnant women without GDM. Levels of miR-29a-3p change significantly over time and between the two groups. Dysregulated miRNA levels during gestation could contribute to decreased insulin sensitivity, an inappropriate response to the increasing insulin resistance associated with pregnancy and, ultimately, to impaired glucose metabolism. Additionally, a higher degree of adiposity has been associated with higher miRNA level. It remains to be identified which cells contribute to the total pool of circulating miRNAs in pregnancy. However, our study highlights the importance of investigating miRNA levels at multiple time points and contributes to a further understanding of GDM pathophysiology.

## Figures and Tables

**Table 1:** Clinical and biochemical parameters at baseline as well as offspring characteristics.

	Normal Glucose Tolerance (NGT)	Gestational Diabetes Mellitus (GDM)	*p*-Value
N	41	41	
Gestation at baseline (weeks)	15.2 ± 2.4	15.3 ± 2.5	0.909
Age (years)	33.2 ± 3.8	32.7 ± 4.0	0.532
Weight (kg)	94.3 ± 12.2	92.6 ± 11.5	0.504
BMI (kg/m^2^)	33.3 (32.2–35.4)	33.3 (31.7–35.9)	0.926
Waist circumference (cm)	107.0 ± 10.7	105.6 ± 8.8	0.501
Neck circumference (cm)	36.0 (35.2–37)	35.5 (34.6–36.9)	0.284
Fat %	30.9 ± 3.0	30.5 ± 2.8	0.623
Fasting glucose (mmol/L)	4.7 (4.3–4.8)	4.9 (4.6–5.2)	**0.003**
1 h glucose (mmol/L)	7.2 (5.9–7.6)	7.2 (6.2–8.3)	0.333
2 h glucose (mmol/L)	5.6 (4.9–6.5)	6.4 (5.7–7.4)	**0.005**
HbA1c (%)	5.0 (4.8–5.4)	5.2 (5–5.5)	0.159
Fasting insulin (μU/mL)	11.9 (9.7–18.4)	14.6 (9.4–18.5)	0.607
1 h insulin (μU/mL)	95.2 (57.5–161.1)	95.8 (52.5–175.5)	0.878
2 h insulin (μU/mL)	52.2 (34.5–88.1)	59.8 (46.9–100.7)	0.145
HOMA-IR index	2.61 (1.98–3.70)	3.11 (2.13–4.19)	0.317
QUICKI index	0.330 ± 0.020	0.325 ± 0.027	0.336
Triglycerides (mmol/L)	1.32 (1.13–1.6)	1.27 (0.9–1.68)	0.517
Free Fatty Acids (mmol/L)	0.61 ± 0.19	0.65 ± 0.17	0.328
HDL cholesterol (mmol/L)	1.46 ± 0.23	1.49 ± 0.26	0.534
LDL cholesterol (mmol/L)	3.16 ± 0.81	3.32 ± 0.75	0.352
Leptin (pg/mL)	36.0 (25.2–46.1)	38.9 (29.5–47.3)	0.799
Ethnicity (European/non-European)	37/4	32/9	0.304
Parity (nullipara/multipara)	18/23	26/15	0.076
Relative with diabetes (no/yes)	33/8	29/12	0.304
Smoking during pregnancy (no/yes)	37/4	36/5	0.724
Offspring sex (male/female)	23/18	20/21	0.507
Birth weight (g)	3548 ± 478	3557 ± 496	0.935

Baseline characteristics for anthropometric and clinical parameters measured during gestation as well as offspring characteristics. Normally distributed data are presented as means ± standard deviations, while non-normally distributed data are presented as medians (interquartile ranges). NGT: Normal glucose tolerance. GDM: Gestational diabetes mellitus, BSA: Body surface area. Continuous data were analyzed either by an independent t-test if data were normal disturbed or a Mann–Whitney test for non-normally distributed data. Categorical data were compared by a chi-square test (2-sided). HOMA-IR: Homeostatic index of insulin resistance, QUICKI: Quantitative insulin sensitivity check index. Fasting, 1 and 2 h glucose were measured in plasma, while fasting, 1 and 2 h insulin were measured in serum. Significant *p*-values (<0.05) are given in bold.

**Table 2:** Relationship between miRNA levels at 24–28 weeks of gestation and gestational weight gain.

	miR-433-3p		miR-134-5p	
	β (95% CI)	*p*	β (95% CI)	*p*
Specific miRNA levels at baseline	0.36 (0.18–0.53)	**0.0002**	0.37 (0.16–0.58)	**0.001**
Weight gain at 24–28 weeks (kg)	0.08 (0.01–0.14)	**0.022**	0.095 (0.03–0.16)	**0.006**
Difference in gestational age (weeks)	−0.01 (−0.08–0.06)	0.722	−0.036 (−0.11–0.03)	0.293
Maternal age at baseline (year)	−0.02 (−0.06–0.03)	0.471	−0.018 (−0.06–0.03)	0.424
Pre-pregnancy BMI (kg/m^2^)	−0.02 (−0.07–0.03)	0.386	−0.005 (−0.05–0.04)	0.834
GDM group	0.27 (−0.06–0.59)	0.104	0.153 (−0.18–0.48)	0.354

Linear regression analysis of miRNAs, which were positively correlated with gestational weight gain. The level of each miRNA at 24–28 weeks of gestation was adjusted for the baseline levels of the specific miRNA, as well as the gestational weight gain from baseline to 24–28 weeks of gestation, maternal pre-pregnancy BMI and age at baseline. Reference group: NGT women. Significant *p*-values (<0.05) are given in bold.

**Table 3:** Relationship between the levels of miR-134 at 35–37 weeks of gestation and changes in HOMA-IR from baseline to 35–37 weeks.

	miR-134-5p at 35–37 Weeks of Gestation	
	β (95% CI)	*p*
Baseline miR-134-5p levels	0.18 (−0.04–0.40)	0.104
Changes in HOMA-IR from baseline to 35–37 weeks	0.07 (0.01–0.12)	**0.030**
Difference in gestational age baseline to 35–37 weeks (weeks)	−0.03 (−0.10–0.04)	0.418
Maternal age at baseline (year)	−0.06 (−0.11–0.02)	**0.010**
Pre-pregnancy BMI (kg/m^2^)	−0.05 (−0.10–0.002)	0.060
GDM Group	−0.09 (−0.44–0.27)	0.620

Linear regression analysis of the changes in insulin resistance (HOMA-IR) from baseline to 35–37 weeks of gestation and its association with the miR-134-5p levels at 35–37 weeks of gestation. Adjustments for maternal age at baseline, as well as differences in gestational age, pre-pregnancy BMI and the miR-134-5p levels at baseline, were made. Reference group: NGT women. Significant *p*-values (<0.05) are given in bold.

**Table 4:** Relationship between the levels of miR-103 at 24–28 weeks of gestation and changes in lipids from baseline to 24–28 weeks.

	miR-103-3p at 24–28 Weeks of Gestation	
	β (95% CI)	*p*
Baseline miR-103-3p	0.18 (−0.03–0.39)	0.092
Changes in LDL cholesterol from baseline to 24–28 weeks (mmol/L)	0.29 (0.04–0.54)	**0.026**
Changes in HDL cholesterol from baseline to 24–28 weeks (mmol/L)	1.39 (0.25–2.53)	**0.017**
Changes in triglycerides from baseline to 24–28 weeks (mmol/L)	−0.01 (−0.49–0.47)	0.967
Difference in gestational age from baseline to 24–28 weeks (weeks)	−0.13 (−0.23–0.03)	**0.010**
Maternal age at baseline (years)	0.01 (−0.05–0.06)	0.814
Pre-pregnancy BMI(kg/m^2^)	−0.01 (−0.08–0.06)	0.800
GDM Group	0.31 (−0.11–0.74)	0.143

Linear regression analysis of the changes in either HDL-, LDL cholesterol or triglycerides from baseline to 24–28 weeks of gestation. Adjustments for maternal age at baseline, as well as differences in gestational age, pre-pregnancy BMI and the miR-103-3p levels at baseline, were made. Reference group: NGT women. Significant *p*-values (<0.05) are given in bold.

## Data Availability

The raw data supporting the conclusions of this manuscript will be made available by the authors, without undue reservation, on request to the corresponding author.

## Supplementary Materials

The following supporting information can be downloaded at: https://www.mdpi.com/article/10.3390/biomedicines10020482/s1, Figure S1: Temporal profile of selected anthropometric variables, Figure S2: Correlations between clinical variables and the selected miRNA at each separate time point during gestation, Figure S3: Comparison between predicted targets of the selected miRNAs and genes highly enriched in human placenta, Table S1: Primers used in the current study.

## Members of the DALI Core Investigator Group

Gernot Desoye (Project Coordinator, Austria, gernot.desoye@medunigraz.at), David Simmons (Trial Coordinator, UK, Da.Simmons@westernsydney.edu.au), Rosa Corcoy (Spain, RCorcoy@santpau.cat), Juan M. Adelantado Perez (Spain, adelantadojm@gmail.com), Alexandra Kautzky-Willer (Austria, alexandra.kautzky-willer@meduniwien.ac.at), Jürgen Harreiter (Austria, juergen.harreiter@meduniwien.ac.at), Peter Damm (Denmark, pdamm@dadlnet.dk), Elizabeth Mathiesen (Denmark, elisabeth.reinhardt.mathiesen@regionh.dk), Dorte M. Jensen (Denmark, Dorte.Moeller.Jensen@rsyd.dk), Lise Lotte T. Andersen (Denmark, lise.lotte.andersen@rsyd.dk), Fidelma Dunne (Ireland, fidelma.dunne@nuigalway.ie), Annunziata Lapolla (Italy, annun-ziata.lapolla@unipd.it), Maria G. Dalfra (Italy, mariagrazia.dalfra@aulss6.veneto.it), Alessandra Bertolotto (Italy, alessandrabertolotto1959@yahoo.it), Mireille van Poppel (The Netherlands, mireil-le.van-poppel@uni-graz.at), Judith G.M. Jelsma (The Netherlands, j.jelsma@amsterdamumc.nl), Sander Galjaard (The Netherlands, s.galjaard@erasmusmc.nl), Ewa Wender-Ozegowska (Poland, ewaoz@post.pl), Agnieszka Zawiejska (Poland, agazaw@post.home.pl), David Hill (Lawson, Canada, David.Hill@lawsonresearch.com), Roland Devlieger (Belgium, ro-land.devlieger@uzleuven.be) and Frank J. Snoek (The Netherlands, fj.snoek@amsterdamumc.nl).
