# Supplementary material for: The Temporal Profile of Circulating miRNAs during Gestation in Overweight and Obese Women with or without Gestational Diabetes Mellitus
Source: Biomedicines. 2022 Feb 18;10(2):482. doi: 10.3390/biomedicines10020482 (PMC8962411; doi:10.3390/biomedicines10020482)
Supplement: Supplementary file 1 [file biomedicines-10-00482-s001.zip › biomedicines-1600397-supplementary.pdf]

biomedicines

MDPI

Original Article – Supplementary Data

The Temporal Profile of Circulating miRNAs During Gestation in Overweight and Obese Women with or without Gestational Diabetes Mellitus

Anja Elaine Sørensen [1]*, Mireille N.M. van Poppel [2], Gernot Desoye [3,4], David Simmons [8], Peter Damm [4], Dorte Møller Jensen [5,6,7], Louise Torp Dalgaard [1], and the DALI Core Investigator Group [9]

Supplementary Figure S1. Temporal profile of selected anthropometric variables

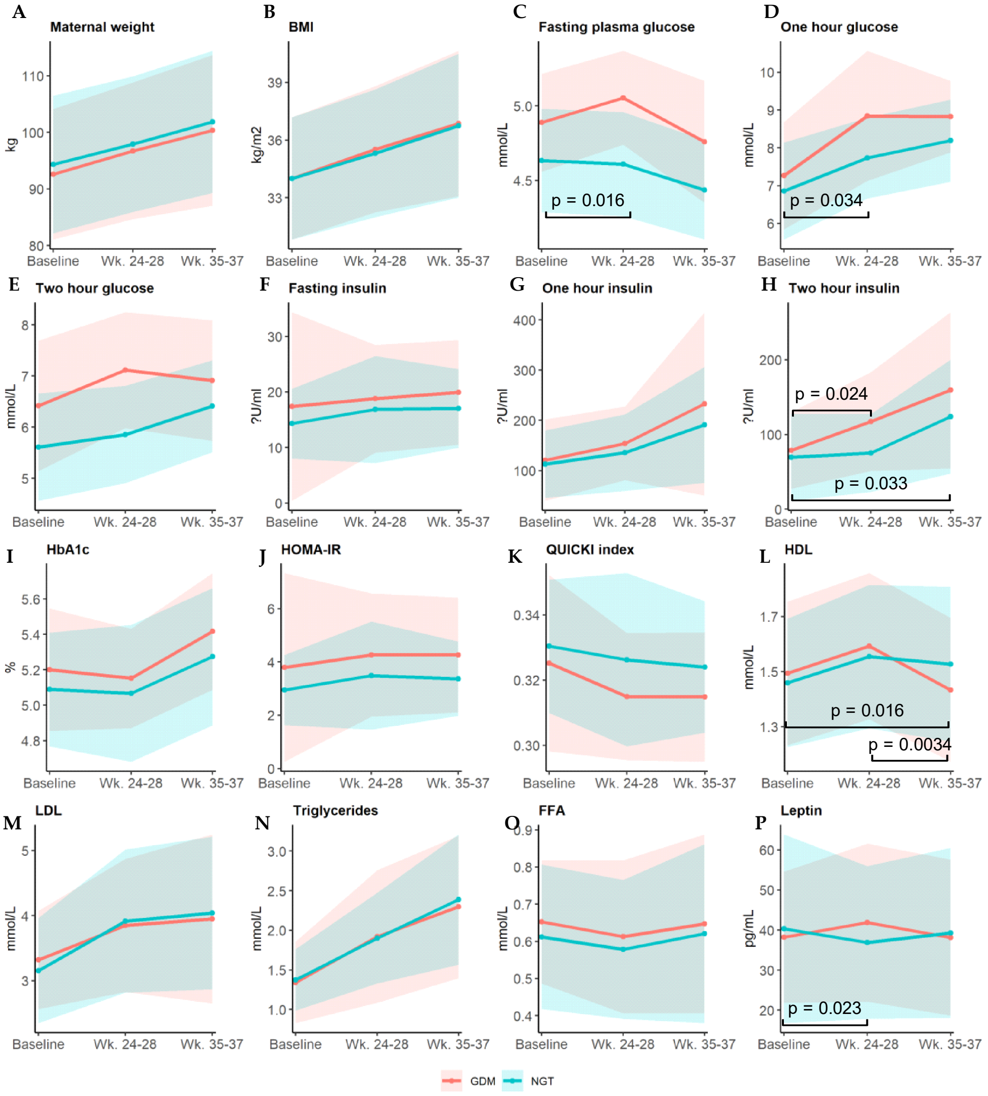

Supplementary Figure S1: Temporal profile of clinical and biochemical parameters during gestation (**A–P**). Shown are mean and standard deviation as ribbons. Women with gestational diabetes (GDM) are depicted in red, while women with normal glucose tolerance (NGT) are shown in blue. At 35-37 weeks, values pertaining to the OGTT (here 1h- and 2h glucose and insulin) are based on women with GDM based on centrally measured glucose values (n = 25). Wk.: Weeks. P-values are based on mixed linaer models as described in the Material and Methods section, and shown are the significant interactions.

Supplementary Figure S2. Correlations between clinical variables and the selected miRNA at each separate time point during gestation.

A

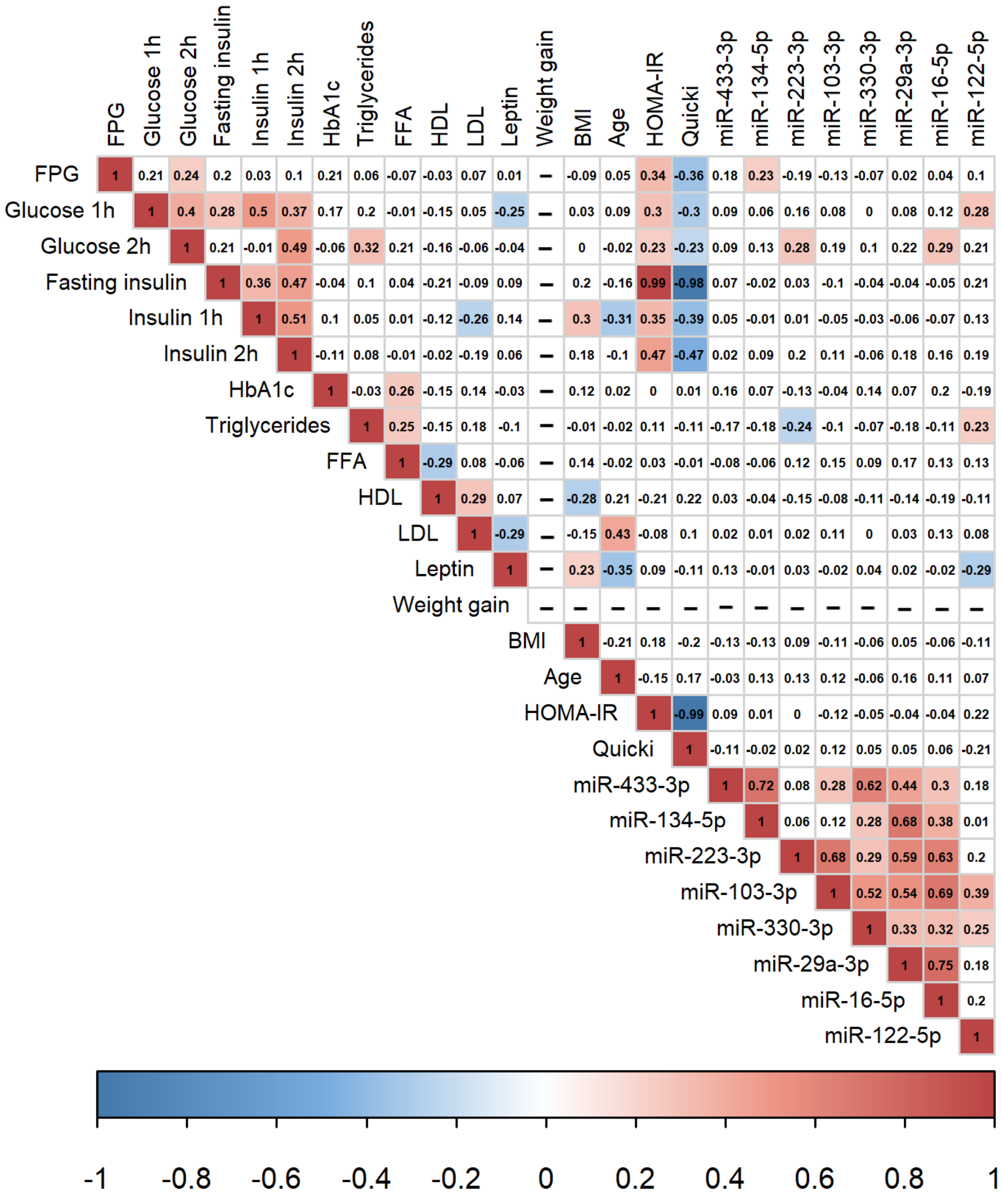

B

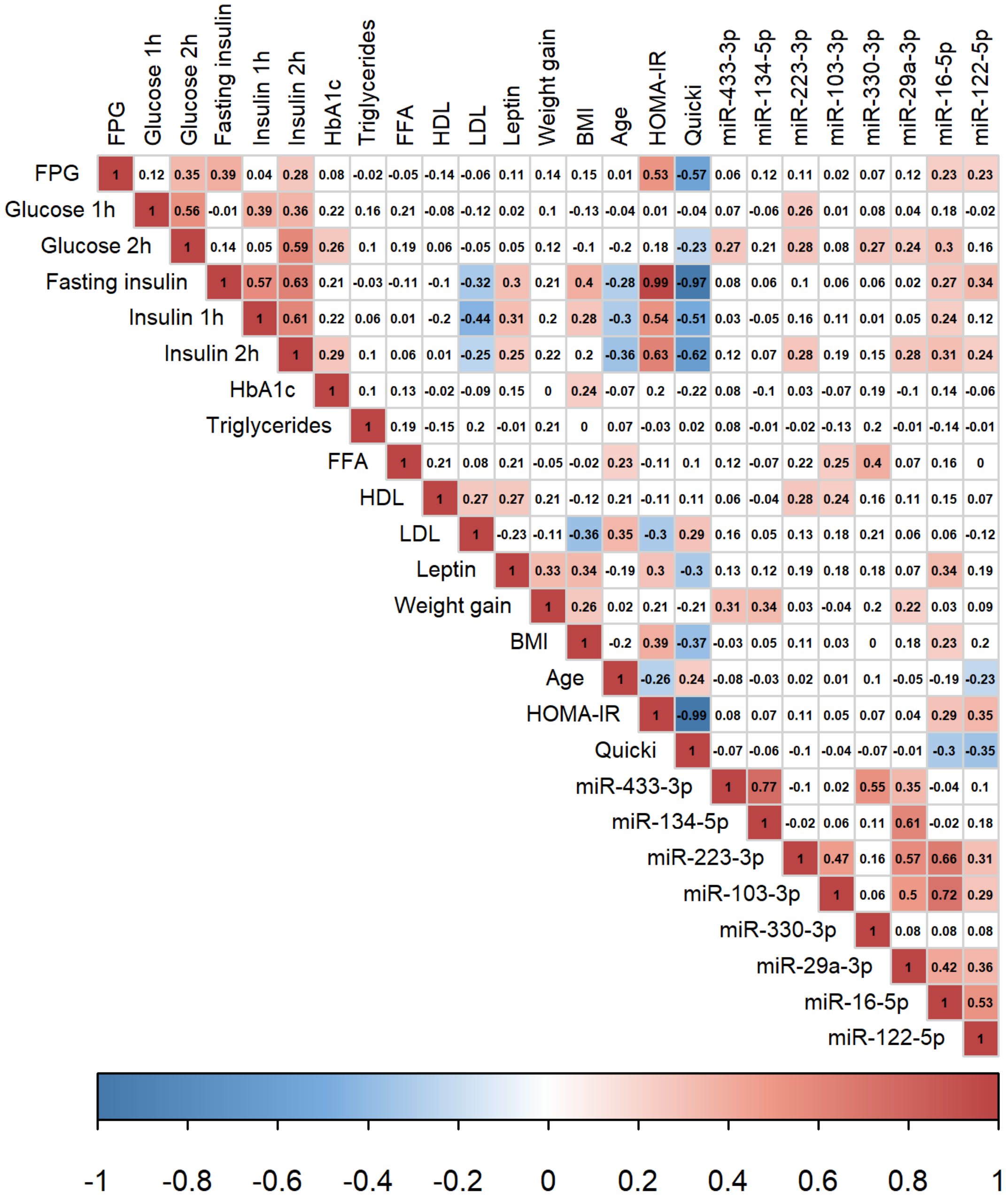
FPG
Glucose 1h
Glucose 2h
Fasting insulin
Insulin 1h
Insulin 2h
HbA1c
Triglycerides
FFA
HDL
LDL
Leptin
Weight gain
BMI
Age
HOMA-IR
Quicki
miR-433-3p
miR-134-5p
miR-223-3p
miR-103-3p
miR-330-3p
miR-29a-3p
miR-16-5p
miR-122-5p
-1
-0.8
-0.6
-0.4
-0.2
0
0.2
0.4
0.6
0.8
1

C

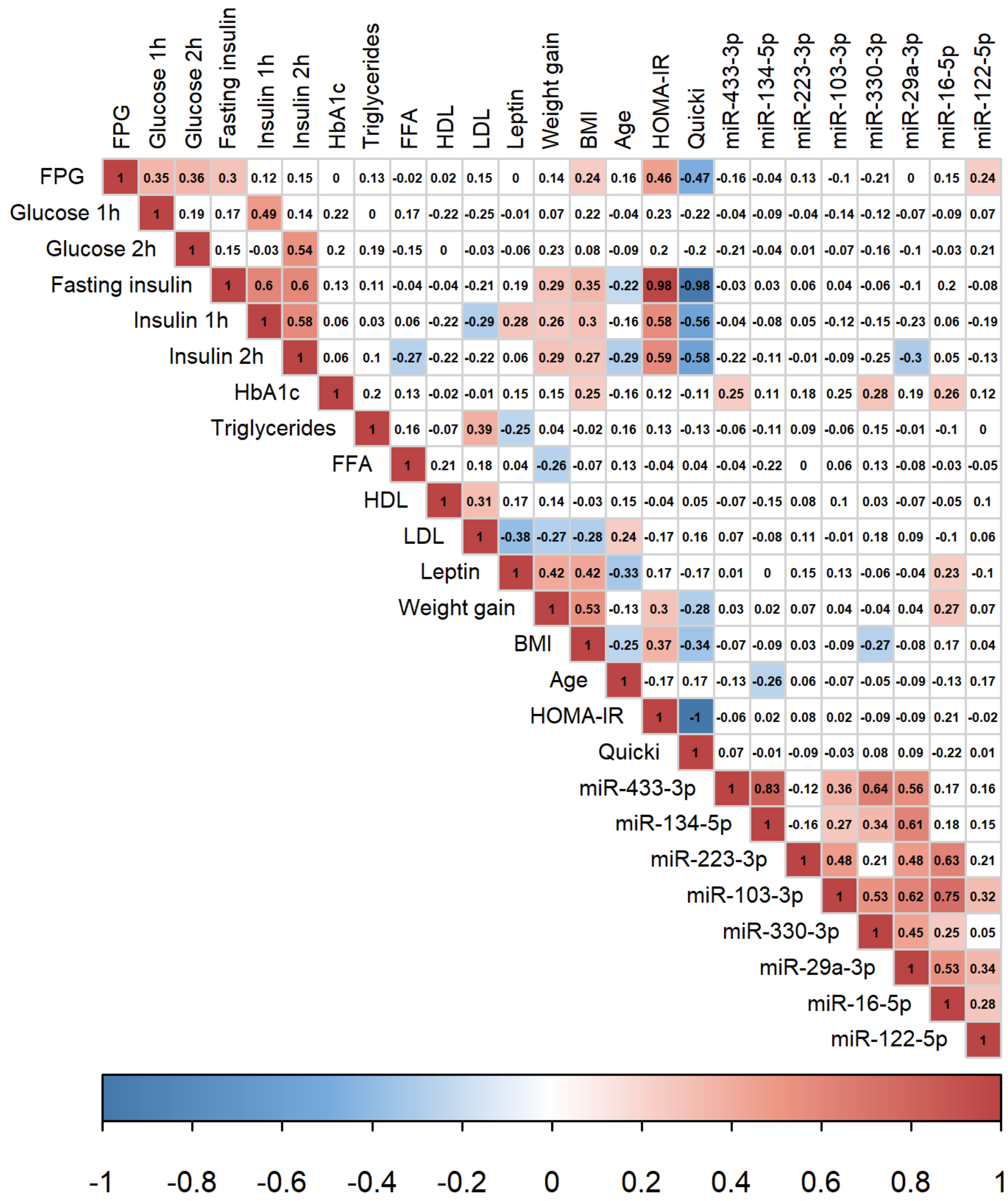

Supplementary Figure S2: Correlation between the selected miRNAs and clinical parameters at each separate time point. (**A**) Baseline, (**B**) 24-28 gestational week and (**C**) 35-37 gestational week. Color intensity is proportional to the Pearson correlation coefficient. Positive correlations are displayed in red and negative correlations in blue colors. Colored correlations are significant at $p<0.05$. No gestational weight gain was present at baseline, here marked with '-'. At 35-37 weeks, corre-lations between a miRNA and values pertaining to the OGTT (here 1h- and 2h glucose and insu-lin) are based on women with GDM based on centrally measured glucose values (n maximum = 25), and women with NGT Levels of miRNAs were normalized against the geometric mean of snRNA U6, ath-miR-159 and c.el.miR-39 and logarithmically transformed prior to analysis as were selected clinical variables according to the methods section.

Supplementary Figure S3. Comparison between predicted targets of the selected miRNAs and genes highly enriched in human placenta.

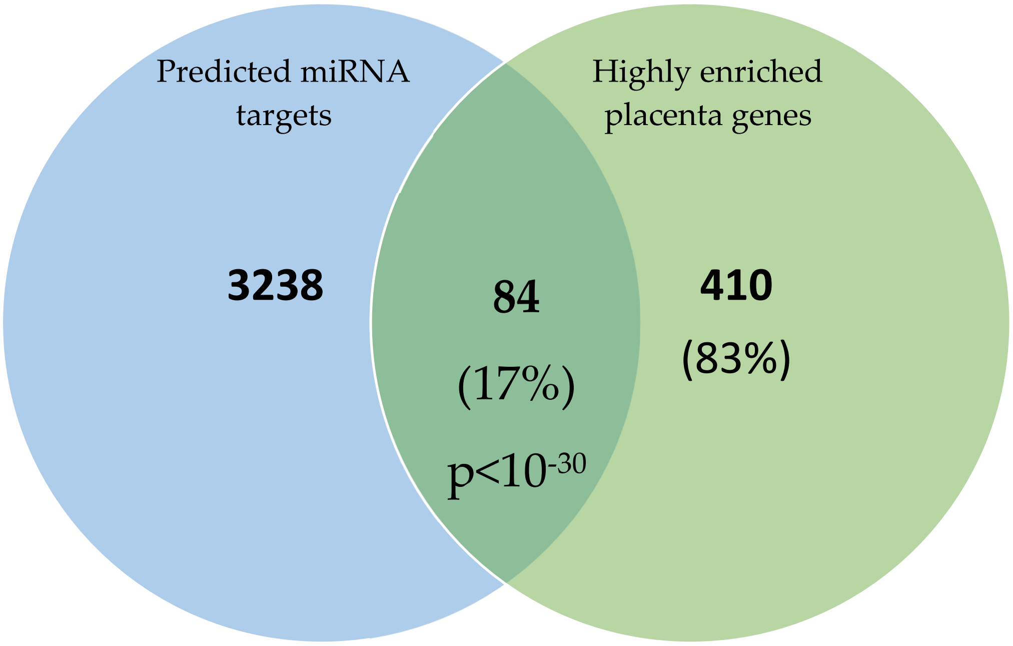

Supplementary Figure S3: Venn diagram representing the number of predicted targets (n=3322) of the selected miRNAs and the number of highly enriched genes (n=494) found in human placenta based on the Human Protein Atlas. The statistical significance of the overlap between two groups is calculated based on exact hypergeometric probability.

Supplementary Table S1. Primers used in the current study

Name	Link to GDM and/or Purpose	Forward 5′-3′<br>Reverse 5′-3′	Ref.
c.el-miR-39	Spike-in at cDNA synthesis	ACACTCCAGCTGGGTCACCGGGTGTAAATC<br>CTCAACTGGTGTCGTGGAGTCGGCAATTCAGTT-GAGCAAGCTGA	
U6	Endogenous reference gene	CTCGCTTCGGCAGCACA<br>AACGCTTCACGAATTTGCGT	
Universal Reverse primer (UPR)	Applied in most assays as universal reverse primer	TGGTGTCGTGGAGTCG	(Chen et al. 2008)
at-miR-159a	Spike-in at RNA isolation	ACACTCCAGCTGGG TTTGGATTGAAGGG<br>CTCAACTGGTGTCGTGGAGTCGGCAATTCAGTT-GAGTAGAGCTCC	
miR-223-3p	Up-regulated in GDM	ACACTCCAGCTGGGTGTCAGTTTGTCAAAT<br>CTCAACTGGTGTCGTGGAGTCGGCAATTCAGTT-GAGTGGGGTAT	(Wander et al. 2017; Yoffe et al. 2019)
miR-16-5p	Up-regulated in GDM	ACACTCCAGCTGGG TAGCAGCACGTAAAT<br>CTCAACTGGTGTCGTGGAGTCGGCAATTCAGTT-GAG CGCCAATA	(Cao et al. 2017; Martínez-Ibarra et al. 2019; Zhu et al. 2015)<br>Sørensen 2021
miR-122-5p	Down-regulated in GDM<br>Dysregulated in metabolic syndrome	ACACTCCAGCTGGG TGGAGTGTGACAATG<br>CTCAACTGGTGTCGTGGAGTCGGCAATTCAGTT-GAG CAAACACC	(Carreras-Badosa et al. 2015)
miR-29a-3p	Dysregulated in GDM	ACACTCCAGCTGGGTAGCACCATCTGAAAT<br>CTCAACTGGTGTCGTGGAGTCGGCAATTCAGTT-GAGTAACCGAT	(Martínez-Ibarra et al. 2019; Wander et al. 2017; Zhao et al. 2011); Sørensen 2021
miR-433-3p	Located in DLK1-DIO3 locus	ACACTCCAGCTGGG ATCATGATGGGCTCC<br>CTCAACTGGTGTCGTGGAGTCGGCAATTCAGTT-GAG ACACCGAG	
miR-134-5p	Located in DLK1-DIO3 locus AND Up-regulated in GDM	ACACTCCAGCTGGG TGTGACTGGTTGACC<br>CTCAACTGGTGTCGTGGAGTCGGCAATTCAGTT-GAG CCCCTCTG	(Strutz et al. 2018)<br>Sørensen 2021
miR-330-3p	Up-regulated in GDM	ACACTCCAGCTGGG GCAAAGCACACGGCCT<br>CTCAACTGGTGTCGTGGAGTCGGCAATTCAGTT-GAG TCTCTGCA	(Martínez-Ibarra et al. 2019; Sebastiani et al. 2017)
miR-103-3p	Dysregulated in pregnancy	ACACTCCAGCTGGG AGCAGCATTGTACAGG<br>CTCAACTGGTGTCGTGGAGTCGGCAATTCAGTT-GAG TCATAGCC	(Adaikalakoteswari et al. 2017; Carreras-Badosa et al. 2015)
